# Clinical study of total bone marrow combined with total lymphatic irradiation pretreatment based on tomotherapy in hematopoietic stem cell transplantation of acute leukemia

**DOI:** 10.3389/fonc.2022.936985

**Published:** 2022-08-11

**Authors:** Fanyang Kong, Shuaipeng Liu, Lele Liu, Yifei Pi, Yuntong Pei, Dandan Xu, Fei Jia, Bin Han, Yuexin Guo

**Affiliations:** Department of Radiation Therapy, First Affiliated Hospital of Zhengzhou University, Zhengzhou, China

**Keywords:** acute leukemia, total bone marrow and lymphatic irradiation, tomotherapy, acute toxicity, GvHD

## Abstract

**Objective:**

Allogeneic hematopoietic stem cell transplantation (allo-HSCT) is an effective method for the treatment of refractory and relapsed acute leukemia, and the preconditioning methods before transplantationis one of the important factors affecting the survival of patients. Radiotherapy combined with chemotherapy is the most commonly used preconditioning method before transplantation. This study evaluated the safety and efficacy of total bone marrow combined with total lymphatic irradiation as a preconditioning method before hematopoietic stem cell transplantation.

**Methods:**

Seventeen patients with acute leukemia who were admitted to our center from 2016 to 2020 were selected. The median age was 17 years (8-35). The target area for TMLI includes the total bone marrow and total lymphatic space, and the organs at risk include the lens, lungs, kidneys, intestine, heart, and liver. The patients received a total bone marrow and lymphatic irradiation preconditioning regimen, the related acute adverse reactions were graded, and the prognosis of the patients after transplantation was observed.

**Results:**

During patient preconditioning, only grade 1-2 toxicity was observed, and grade 3-4 toxicity did not occur. Except for one patient whose platelets were not engrafted, all the other patients were successfully transplanted. The median time of neutrophil implantation was 14 d (9-15 d), and the median time of platelet implantation was 14 d (13-21 d). With a median follow-up of 9 months (2-48), 4 relapses occurred, 3 died, and 10 leukemia patients survived and were disease-free. One-year overall survival was 69.8%, cumulative recurrence was 19.5%, disease-free-survival was 54.2%.

**Conclusion:**

The Allo-HSCT pretreatment regimen of total bone marrow combined with total lymphatic irradiation is safe and effective in the treatment of malignant hematological diseases. Total bone marrow combined with total lymphatic irradiation may completely replace total body irradiation, and the clinically observed incidence of acute toxicity is not high.

Hematopoietic stem cell transplantation (HSCT) is one of the main treatments for leukemia, and pretreatment regimen before transplantation is an important factor affecting the survival of patients after transplantation. Total body irradiation (TBI) is usually combined with chemotherapy as a pretreatment regimen for bone marrow transplant patients. TBI can act on certain organs that cannot be reached by chemotherapy drugs to eliminate leukemia cells, and can also suppress the patient’s immune function so that the donor’s hematopoietic stem cells can be successfully engrafted (1, 2).

However, due to the lack of accuracy and organ targeting of TBI, high radiation doses will involve normal tissues or organs (lung, heart, liver and kidney), leading to radiation-related toxicity and increased transplant-related mortality (TRM). There are many potential complications of TBI, acute and subacute side effects include nausea, dry mouth, oral mucositis, and interstitial pneumonia (IP); long-term side effects may include venous occlusive disease, neurocognitive impairment, heart disease, cataracts, and secondary tumors (3, 4).

If the prescribed dose of a single treatment is lower than 6 Gy, the risk of transplant failure and post-transplant recurrence is greatly increased, and the incidence of interstitial pneumonia increases significantly when the single dose is increased to more than 10 Gy (5). Fractional irradiation patterns of 12 to 15 Gy had no higher side effects than a single 10 Gy treatment, and treatment mortality was reduced (6). The more common pattern of fractional TBI was 12Gy/6F (7).

Due to concerns about late toxicity, the use of TBI is gradually decreasing as part of the preconditioning methods before transplantation for patients with acute leukemia. Total marrow irradiation (TMI) and total bone marrow combined with total lymphatic irradiation (TMLI) as part of tomotherapy is a more targeted approach to radiation therapy, which can prioritize the delivery of doses to areas with a high tumor burden while reducing toxicity and increasing the dose that is projected to the bone marrow (8–10).

In the pretreatment of leukemia patients, the optimal irradiation dose of TMLI and combination chemotherapy are still under exploration. Because TMI/TMLI is in the exploratory stage of development, it has not yet been used as a standard pre-transplant component. This study mainly evaluated the safety and efficacy of total bone marrow combined with total lymphatic irradiation as a preconditioning method before allogeneic peripheral blood stem cell transplantation.

## Materials and methods

### Patients in the group

The functions of the heart, liver and kidney were basically normal, and the KPS score was ≥ 80. The clinical diagnosis is clear and indicates bone marrow transplantation. As shown in **Table 1**, a total of 17 patients with TMLI were selected from January 2016 to March 2020, including 15 males and 2 females, aged 8 to 35 years (median 17 years). There were 9 patients with acute B lymphoblastic leukemia, 7 patients with acute T lymphoblastic leukemia and 1 patient with acute myeloid leukemia.

**Table 1:** Patient introduction.

Patient No.	Gender	Age	Diagnosis
1	Male	16	B-ALL
2	Male	12	T-ALL
3	Male	18	B-ALL
4	Male	14	B-ALL
5	Male	10	B-ALL
6	Male	17	B-ALL
7	Male	8	B-ALL
8	Female	10	B-ALL
9	Male	12	T-ALL
10	Male	11	T-ALL
11	Male	35	AML
12	Male	22	T-ALL
13	Male	12	T-ALL
14	Female	16	B-ALL
15	Male	9	B-ALL
16	Male	10	T-ALL
17	Male	25	T-ALL

### Equipment and CT positioning

Tomo HD integrates the X-ray beam of 6 MeV and the CT image guidance function of 3.5 MeV in a single device and can modulate tumors with lengths of 135 cm and widths of 60 cm. Before the CT scan, the patient was fixed in a vacuum pad that covered the whole body, and a thermoplastic mold of the head, neck and shoulder mold was used in combination with the body vacuum pad in the supine position. A 1-cm tissue compensation membrane was placed in the patient’s hands, feet and ribs to ensure that the built-up area was formed. The knee was bent slightly to minimize bending of the lumbar spine. Patients whose height is less than 135 cm underwent CT that scanned, in an advanced manner, all areas from the top of the head to the toes. For patients who are taller than 135 cm, metal marks are placed in the middle of the patient’s thighs, and CT scans are performed in two segments. The first segment is from the top to the lower thigh, the head is advanced, and the layer thickness is 5 mm. The other part removes the thermoplastic mold of the head, neck and shoulder. From the toe to the upper thigh, the foot is advanced, the layer thickness is 5 mm, and the two segments overlap approximately 20 cm. Multiple cross-sectional lines were drawn on all the limbs, and the corresponding position was drawn on the vacuum pad so that the position was more accurate during treatment.

### Target delineation

**Figure 1:**
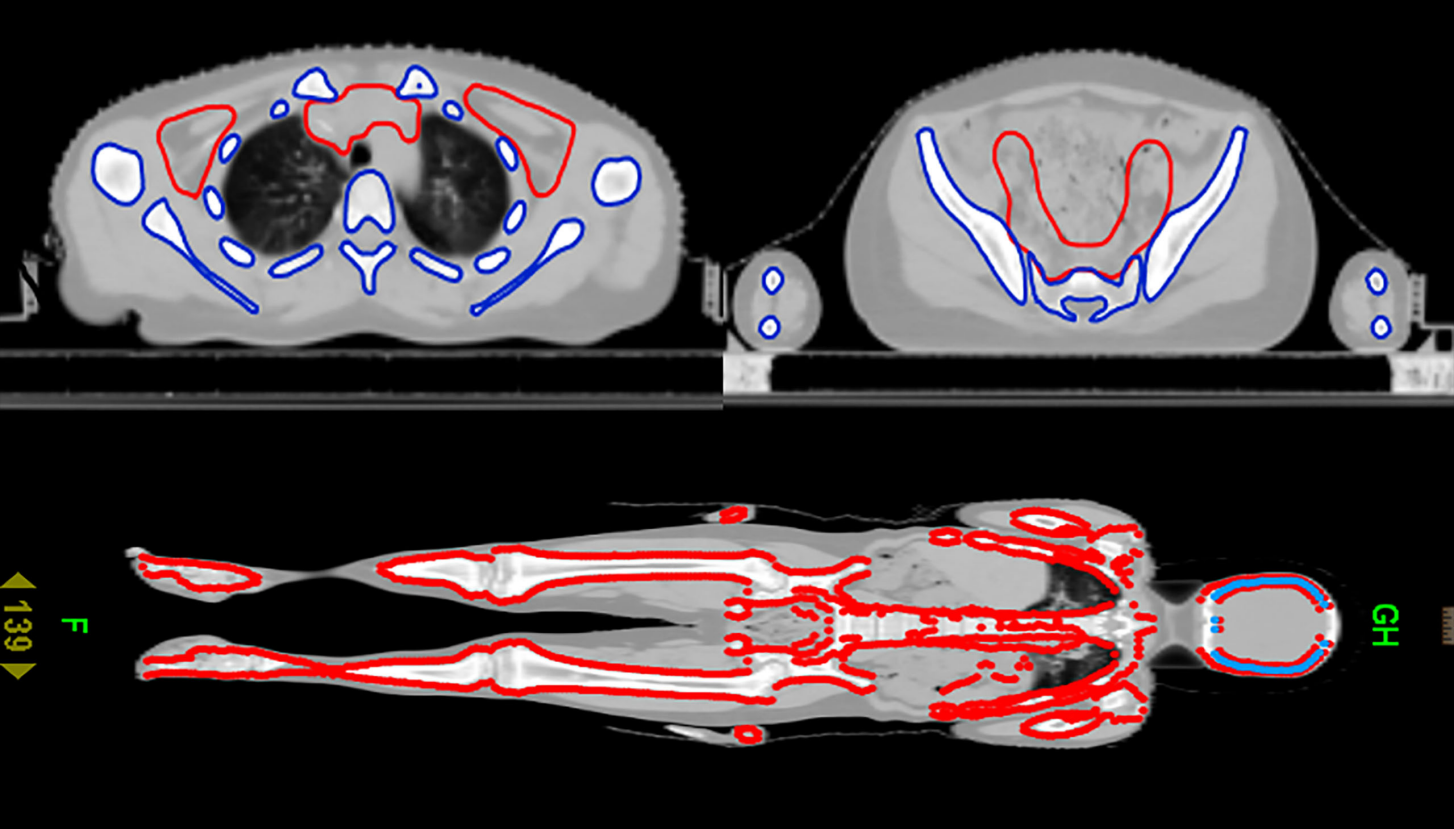
Cross-sectional and coronal display of the TMLI target.

After CT scanning, the data were transmitted to the Varian TPS planning system for target and organ delineation. TBI target definition: subtract all human tissues from the lens and bilateral lungs. The target area of TMLI is defined as total bone marrow and all lymph nodes, including the spleen, brain and testes. Total bone marrow includes skull, mandible, humerus, scapula, clavicle, sternum, vertebra, rib, hip, femur, limb bone, etc. Lymph nodes include cervical lymph nodes, mediastinal lymph nodes, supraclavicular lymph nodes, and inguinal lymph nodes, etc. Organs at risk include the lens, lung, heart, kidney, intestine, liver, etc. The target area for TMLI is delineated, as shown in **Figure 1**. A CTV uniformly expanded by 5 mm is defined as the PTV.

### Plan design

TBI and TMLI plans based on Tomo TPS were designed for the patients in the group. The Field Width is 5.054 cm, the Pitch is 0.287, and the modulation factor is 3. The Dose Calc Grid is Fine mode 1.95 mm. For lower limb MF=2.0, the other parameters are consistent. The PTV prescription was 12 Gy/6 F, and TBI and TMLI plans were normalized to 95% of the prescription dose volume. Each patient plans to iterate 300 times, and it takes approximately 15 hours for each plan to optimize the modulation time. However, the patients actually adhered to the TMLI plan, as shown in **Table 4**. The treatment was administered twice a day with an interval of 8 hours.

### Efficacy evaluation indicators after transplantation

Hematopoietic reconstruction indicators, that is, the time of granulocyte and platelet engraftment (the first day of neutrophil implantation is defined as neutrophil implantation time when neutrophils ≥0.5×10^9^/L for 3 consecutive days, and platelet implantation time is defined as platelet implantation time when neutrophils are counted for 7 consecutive days ≥ 20×10^9^/L without blood transfusion); all patients were graded for preconditioning-related acute adverse reactions; the occurrence of GVHD after transplantation and the prognosis after transplantation were observed.

### SPSS 26.0 was used for statistical analysis

R software was used to plot Kaplan-Meier survival curve and one-year OS, CIR, DFS estimates, and log-rank test was used to compare whether there were statistical differences between different survival curves. The test level is α = 0.05, and the difference is considered to be statistically significant when the P<0.05 is performed.

## Results

**Figure 2:**
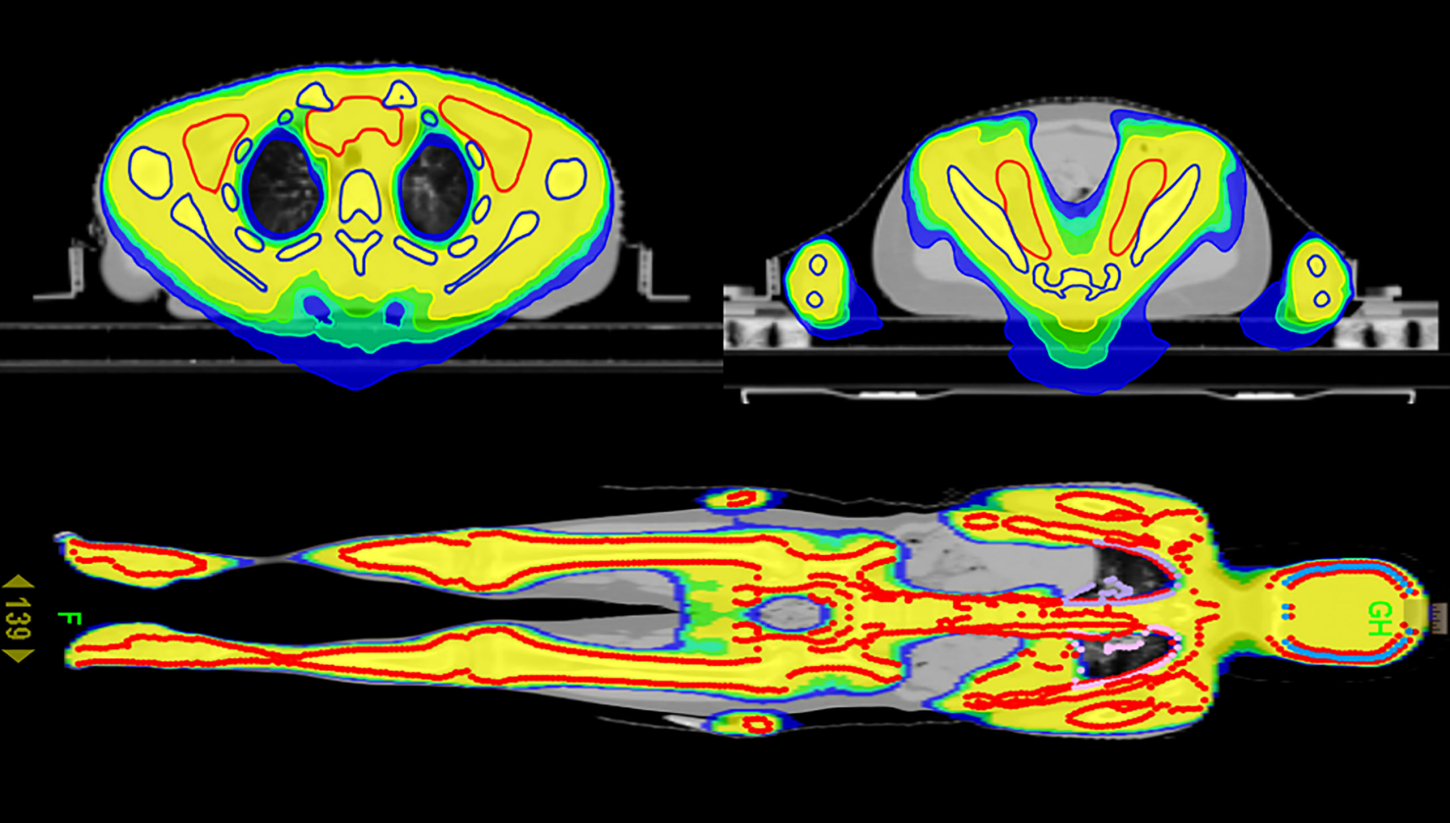
Cross-sectional dose distribution and coronal dose distribution of TMLI based on HT (12 Gy).

As shown in **Table 2**, compared with the TBI regimen, the DVHs of the TMLI regimen with the same dose showed that almost all the important organs at risk had varying degrees of risk reduction. The TMLI regimen reduced the average dose administered to organs by 15.0% to 57.6%. The average doses administered to the lungs, liver, heart, intestine and stomach decreased by 17%, 45.1%, 52.9%, 40.7% and 55.3%, respectively. The average treatment time of TBI was 32.4 min. The average treatment time of TMLI was 29.9 min. The cross-sectional dose distribution and coronal dose distribution of TMLI based on HT (12 Gy) were shown in **Figure 2**.

**Table 2:** Comparison of the average dose of TBI and TMLI endangered organs (12 Gy).

Organs at risk (OARs)	TBI Dose (Gy) Mean	TMLI Dose (Gy) Mean	Average Dose Reduction (%)
Left Lens Right Lens Left Eye Right Eye Left Parotid Right Parotid Heart Left Lung Right Lung Left Kidney Right Kidney Stomach Liver Intestine	3.94 3.72 8.61 8.56 12.78 12.75 12.00 7.36 7.32 12.69 12.71 12.44 12.44 12.47	3.18 3.15 5.47 5.46 5.93 5.72 5.65 6.11 6.08 5.40 5.39 5.56 6.83 7.40	19.3% 15.3% 36.5% 36.2% 53.6% 55.1% 52.9% 17.0% 16.9% 57.5% 57.6% 55.3% 45.1% 40.7%
Body	12.24	10.4	15.0%

Every patient received two MVCT images with which to perform image-guided treatment, and the corresponding MVCT scan areas were the head-to-chest area and the pelvic area. Each image was registered with a corresponding kilovoltage CT (kVCT) image. If the pitch, yaw or roll were more than 2° or the offset of X, Y or Z was more than 5 mm, the positioning was redone. MVCT was acquired again to ensure that the pitch, yaw and roll were less than 2°, and the offsets of X, Y and Z were less than 5 mm. The average value of the offset of the two positions was taken as the final registration result. Once accepted, the examination table was moved to the registration position to begin the treatment.

**Figure 3:**
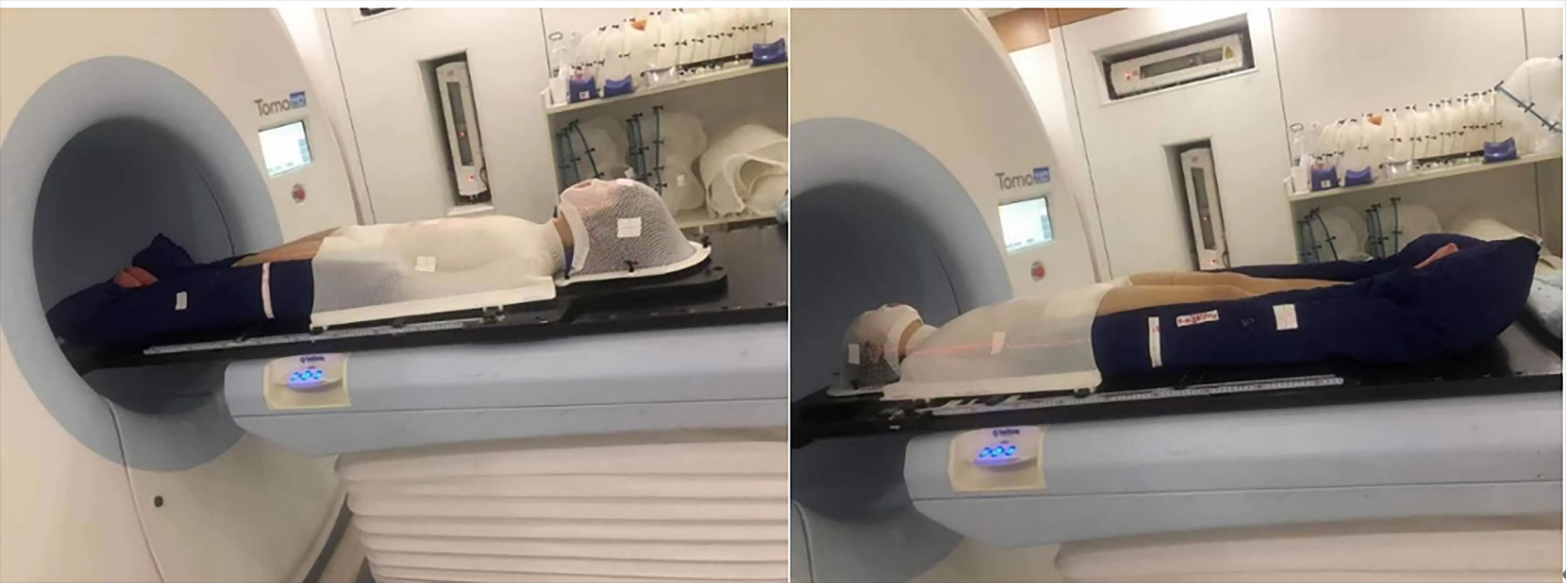
The upper section head advanced mode and the lower foot advanced mode.

As shown in **Figure 3**, if the patient is taller than 135cm, the treatment was carried out in two stages, including the upper section head advanced mode and the lower foot advanced mode.

As shown in **Table 3**, 17 patients were classified into regimen-related toxicity according to organ system (11). Toxicity was most common in the Mucosa and Gut. Only grade I-II toxicity was observed, and grade III-IV toxicity did not appear. After symptomatic treatment with antiemetic drugs, antidiarrheal drugs, rehydration drugs, etc., all patients tolerated it. Mild toxic reactions occurred during pretreatment, and no radiation pneumonia and hepatic radiation-induced veno-occlusive occurred.

**Table 3:** Regimen-related toxicity according to organ system.

	Grade 0		Grades I or II		Grades III or IV	
	n	%	n	%	n	%
Heart	17	100	0	0	0	0
Bladder	17	100	0	0	0	0
Kidneys	17	100	0	0	0	0
Lungs	17	100	0	0	0	0
Liver	17	100	0	0	0	0
CNS	17	100	0	0	0	0
Mucosa	15	88.2	2	11.8	0	0
Gut	11	64.7	6	35.3	0	0

All patients were successfully transplanted except one patient whose platelets were not implanted. The median time of neutrophil implantation was 14 d (9~15 d), and the median time of platelet implantation was 14 d (13~21 d).

In **Table 4**, all patients were set to a prescribed dose of 12Gy/6F.Four patients were given only 10Gy/5F due to reasons such as diarrhea and other machine failures. Follow-up observation was conducted after transplantation, as shown in **Table 4**, and the follow-up was performed September 1st, 2021. The median follow-up period was 9 months (2-48 months). Among all patients, 1 patient developed acute graft-versus-host disease, and 2 patients developed chronic graft-versus-host disease. 4 patients experienced recurrence, 3 patients died, and 10 patients with leukemia survived and were disease-free. Except for 1 case of extramyelial recurrence, the others were hematologic recurrence.

**Table 4:** Disease condition of patients before and after transplantation and survival time after transplantation.

Patient No.	Pretransplant state	Transplantation type	Dose (Gy)	Survival time (months)	Survival state
1	CR	Haploidentical	10	20	disease-free
2	CR	Haploidentical	12	21	disease-free
3	NR	Haploidentical	10	3	died
4	CR	Haploidentical	12	10	recurred
5	CR	HLA-matched	10	8	recurred
6	CR	HLA-matched	12	18	disease-free
7	CR	Haploidentical	12	6	disease-free
8	CR	Haploidentical	12	28	recurred
9	CR	Haploidentical	12	5	died
10	CR	Haploidentical	12	6	disease-free
11	CR	HLA-matched	10	6	recurred
12	CR	HLA-matched	12	13	disease-free
13	CR	HLA-matched	12	5	disease-free
14	CR	Haploidentical	12	9	disease-free
15	CR	Haploidentical	12	2	died
16	CR	Haploidentical	12	48	disease-free
17	CR	HLA-matched	12	13	disease-free

**Figure 4:**
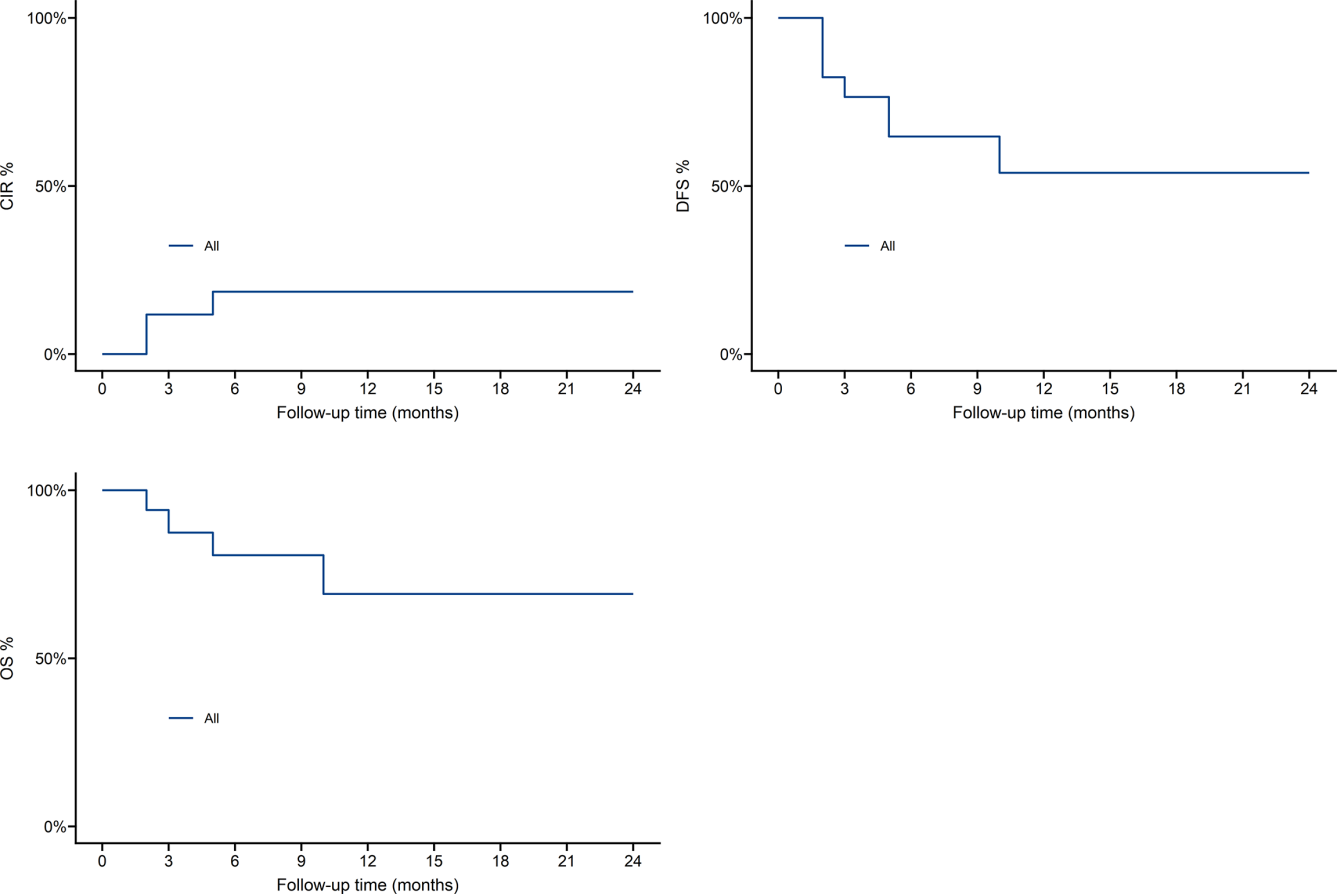
Survival outcomes in 17 patients.

**Figure 5:**
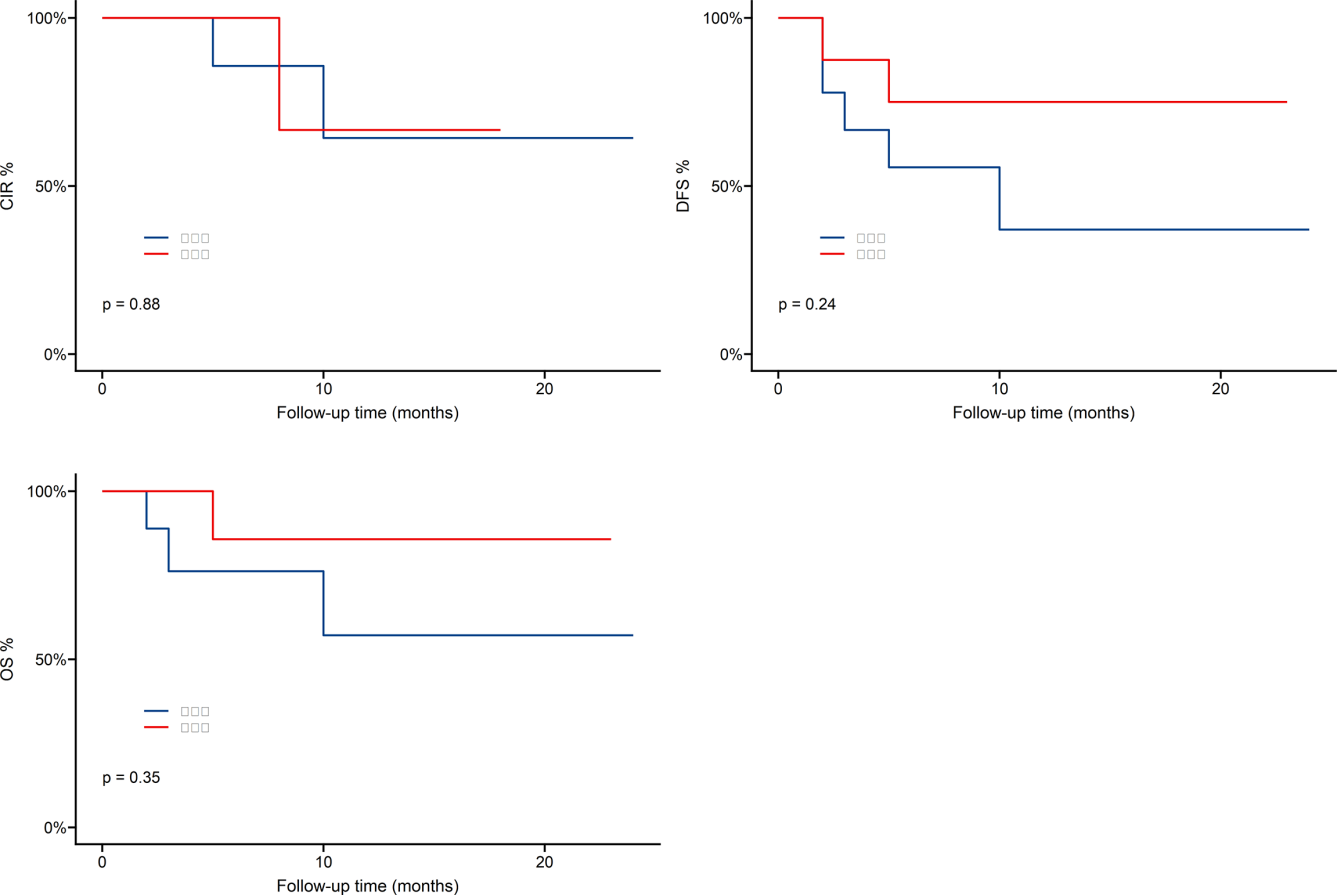
Survival outcomes of Haploidentical versus HLA-matched transplantation.

As shown in **Figure 4**, one-year overall survival (OS) was 69.8%, cumulative incidence recurrence (CIR) was 19.5%, disease-free-survival (DFS) was 54.2%. Patients who received Haploidentical versus HLA-matched transplantation had no statistically significant difference in OS,DFS and CIR, as shown in **Figure 5**.

## Discussion

In the TBI regimen, the main dose limiting factor is pulmonary toxicity. Gruen et al. (7) found that when the average lung dose of the HT-based FTBI 12 Gy/6 F/3 D regimen was 9.14 Gy, no grade 3-4 side effects were observed during 15 months of follow-up. Shinde et al. (12) evaluated hematopoietic cell transplantation in 142 patients with primary multiple myeloma or acute leukemia, and the probability of radiation pneumonia was 0.7% when the average lung dose was kept within 8 Gy. The median dose of left and right lungs in this protocol is 6.1 Gy. Gerstein et al. (13) found that the tolerance dose of the kidney to a fractionated dose of TBI was 14 Gy in adults and 12 Gy in children. The median dose of kidney in this protocol is 5.4 Gy. Hepatic radiation-induced veno-occlusive disease is a complication involving whole liver irradiation. Radiation-induced veno-occlusive disease is almost eliminated by significantly reducing the dose of large volume liver (14). In this protocol, the median dose of liver was reduced by 45.1%, and no hepatic radiation-induced veno-occlusive occurred.

Compared with traditional TBI techniques, Haraldsson et al. (8) studied the helical tomography-based total bone marrow irradiation technique in 23 patients with no increase in GVHD toxicity, recurrence or severity. Whole-body irradiation using helical tomography is feasible and can deliver higher doses to sites at high risk of recurrence while sparing major normal organs such as the lungs, liver, and kidneys, therefore reducing the severity and frequency of late complications (15, 16).

Studies have shown that comparing patients with TMLI and TBI, the rate of extramedullary recurrence after hematopoietic stem cell transplantation is comparable, although TMLI provides patients with more conservative targeted radiation therapy, which does not increase the incidence of extramedullary recurrence risk. Except for 1 case of extramyelial recurrence, the others were hematologic recurrence. Kim et al. (17) studied 101 patients and found that the extramedullary recurrence rate after TMLI was equivalent to the results of the TBI regimen, and there was no increase in the risk of extramedullary recurrence. Wong et al. (18) reported the long-term toxicity of 142 patients who received the TMLI regimen from 2005 to 2016. They believed that the higher dose rate of HT would not cause organ dysfunction. The effect of the dose rate is alleviated by reducing the dose to the organs at risk and changing the fractional exposure pattern.

Hui et al. (19) explained the molecular level changes in mice treated with TBI/TMI and found that the content of stromal cell-derived factor (SDF-1) in the organs or bone marrow of mice treated with TBI increased. The content of SDF-1 in the organs or bone marrow of the latter mice did not increase, which indicated that the donor cells could successfully aggregate into the bone marrow to achieve the goal of successful engraftment. As a chemokine, SDF-1 can cause donor cells to accumulate from the blood to the organ rather than the bone marrow, thus resulting in reduced transplantation efficiency.

Rosenthal et al. (20) proved through experiments that when chemotherapy is combined with TMLI, the intensity of chemotherapy can be appropriately reduced so that patients can obtain transplantation with low toxicity, while the recurrence rate of transplantation does not increase, and the success rate of transplantation is higher. In TMLI, irradiation of the total body skeleton and major lymph nodes can provide an adequate immunosuppressive response to the graft. Existing clinical studies have confirmed that TMLI can reduce the occurrence of graft-versus-host disease (21). In addition, the graft-versus-tumor effect can be preserved (22).

DVH plays an important role in predicting the radiation toxicity of organs (23). Acute complications of absolute logarithmic therapy are caused by the response of these organs, and data from these targeted TMLI schemes can predict a reduction in incidence. In the preliminary design of the TMLI scheme study, only grade 1-2 toxicity was observed, and grade 3-4 toxicity did not appear. There was 1 patient with acute graft-versus-host disease and 2 patients with chronic graft-versus-host disease, of which 4 patients experienced recurrence, 3 patients died and 10 patients with leukemia survived and were disease-free. one-year overall survival was 69.8%, cumulative incidence recurrence was 19.5%, disease-free-survival was 54.2%. As a preconditioning radiotherapy for patients with acute leukemia, TMLI is safe and effective.

For patients with relapsed and refractory leukemia, compared with TBI, the dose of TMI should be increased while ensuring an effective reduction in the recurrence rate without causing a corresponding degree of severe radiotherapy-related toxicity (24, 25). Hui et al. (26) studied that TMI dose escalation to 15 Gy is feasible with acceptable toxicity in pediatric and adult patients with high-risk leukemia undergoing umbilical cord blood and sibling donor transplantation. In the pretreatment of leukemia patients, the optimal irradiation dose of TMI and combination chemotherapy are still under exploration. After accumulating more TMLIs experience, dose escalation is the next step of our radiotherapy center.

## Conclusion

The results of this study suggest that helical tomographic intensity-modulated radiation therapy is clinically feasible for TMLI, TMLI can replace TBI as preconditioning before bone marrow transplantation, and TMI-based conditioning regimens can reduce preconditioning complications compared with TBI. No grade 3-4 toxicity occurred in 12 patients in this study, and no case of interstitial pneumonia or hepatic veno-occlusive disease occurred in the later follow-up. Allogeneic blood stem cell transplantation based on TMLI is a safe and effective method for the treatment of malignant hematological diseases with a wider range of indications and treatment. Due to the limited development time of this treatment plan and the small number of cases, its long-term efficacy is still uncertain, and a large-sample randomized controlled study is needed to further confirm its advantages and make its application prospects more promising.

## Data availability statement

The original contributions presented in the study are included in the article/supplementary material. Further inquiries can be directed to the corresponding author.

## Ethics statement

This study was reviewed and approved by The first affiliated hospital of Zhengzhou University. Written informed consent to participate in this study was provided by the participants’ legal guardian/next of kin.

## Author contributions

FK was in charge of data analysis and thesis writing. SL, LL, YFP, YTP and DX collected literature. FJ and BH were responsible for data verification and analysis. YG directed the writing and revision of the thesis. All authors contributed to the article and approved the submitted version.

## Conflict of interest

The authors declare that the research was conducted in the absence of any commercial or financial relationships that could be construed as a potential conflict of interest.

## Publisher’s note

All claims expressed in this article are solely those of the authors and do not necessarily represent those of their affiliated organizations, or those of the publisher, the editors and the reviewers. Any product that may be evaluated in this article, or claim that may be made by its manufacturer, is not guaranteed or endorsed by the publisher.
